# Orthorexia Nervosa and its association with obsessive–compulsive disorder symptoms: initial cross-cultural comparison between Polish and Italian university students

**DOI:** 10.1007/s40519-021-01228-y

**Published:** 2021-06-02

**Authors:** Anna Brytek-Matera, Susanna Pardini, Justyna Modrzejewska, Adriana Modrzejewska, Paulina Szymańska, Kamila Czepczor-Bernat, Caterina Novara

**Affiliations:** 1 Institute of Psychology, University of Wroclaw, Dawida 1, 50-527 Wroclaw, Poland; 2 Department of General Psychology, University of Padova, Padova, Italy; 3 Faculty of Humanities and Social Sciences, University of Bielsko-Biała, Bielsko-Biała, Poland; 4 Department of Psychology, Katowice Business University, Katowice, Poland

**Keywords:** Orthorexia Nervosa, Obsessive–Compulsive Disorder, Obsessive Belief, Perfectionism, Multi-centered Study

## Abstract

**Abstract:**

There is limited evidence of a link between Orthorexia Nervosa (ON) and Obsessive–Compulsive Disorder (OCD), and no definitive conclusions can be drawn. The interplay between socio-cultural context and ON has been poorly investigated as well. Therefore, the objectives of the present study were: (1) to investigate the differences in ON and OCD symptoms and (2) to assess the relationship between ON and OCD symptoms among university students. Six hundred and sixty-six university students participated in the present study: 286 from Poland and 320 from Italy. No age, gender and marital status differences were identified between two samples of university students. However, on average, Polish university students had a higher Body Mass Index than Italian ones. Our findings showed that Polish students present more problems related to obsessive symptomatology, core beliefs of OCD, perfectionism traits, and a major ON symptomatology than Italian ones. Also, Polish students with a higher level of ON exhibited higher levels of OCD symptoms and parental expectations/parental criticism. While Italian students with a higher level of ON showed higher levels of perfectionism features (organization and concern over mistakes). In general, correlations were low as confirmation of partial independence ON from OCD symptoms and core beliefs of OCD in both Polish and Italian university students. The present results highlight a need for further investigation of the correlates of ON across different cultural groups. Future research may screen individuals with ON to determine the comorbidity between ON and OCD symptomology to facilitate appropriate treatment choices.

**Level of evidence:**

Level V, Opinions of respected authorities, based on descriptive studies, narrative reviews, clinical experience, or reports of expert committees.

## Introduction

Healthy eating and healthy lifestyles are deemed to be desirable in Western societies [1, 2], with an increasing emphasis on eating good quality or “clean” foods [3]. For some individuals, the pursuit of an “extreme dietary purity” may become obsessive and lead to orthorexic behaviors [4]. Orthorexia Nervosa has been defined in the literature as obsession or fixation or concern/preoccupation [5] with healthy food consumption and disturbing thoughts, excessive worrying, and rigid, compulsive eating behaviors regarding healthy dietary intake, e.g., avoidance of foods considered “unhealthy” or “unclean” [6]. In the light of ON literature, the use of terms concern/preoccupation, obsession, and fixation seems to be complementary since they deal with different aspects of the same problem: the concern about healthy diet results in the attention captured by food, thus evolving to a persistent and disturbing thought and stereotyped behavior [5].

One of the proposals for diagnostic criteria [7] suggests two critical features of ON: (a) obsessive focus on dietary practices believed to promote optimum well-being through healthy eating with inflexible dietary rules, recurrent and persistent preoccupations related to food, compulsive behaviors, and (b) consequent, clinically significant, impairment, e.g., medical, physical or psychological complications, significant weight loss, malnutrition, extreme emotional distress with feelings of guilt, shame, anxiety, and impairment in critical areas of functioning. Bratman [8], the author who coined the term ON, has revealed orthorexic behavior is only becoming pathological if obsessive thinking, compulsive behavior, self-punishment, and escalating restriction are presented and become central drivers of life while impeding other important areas take place.

It is still debatable whether ON should formally be recognized as a distinct and separate psychiatric diagnosis: an eating disorder—an antecedent of anorexia nervosa or a way to maintain AN or a consequence of AN, a form of the obsessive–compulsive disorder (OCD), or assimilated into the spectrum of an already established psychiatric diagnosis [9–12] or merely a new lifestyle phenomenon, a culturally influenced attitude, rather than a disease [8, 13]. It is worth adding that anecdotal reports of consequences of ON, e.g., fatigue and emotional instability, social isolation, reduced quality of life and physical, malnutrition, and weight loss, follow current concepts of mental disorders [14].

### Orthorexia Nervosa and obsessive–compulsive disorder: comparison

Based on the pattern of excessive preoccupation with healthy eating coexistent with features of obsessive–compulsive personality, diagnosis of ON was first proposed in 2004 by Donini et al. [15]. Asil and Sürücüoğlu [16] found that dietitians with ORTO-15 scores less than 40 points showed higher OCD symptoms. Similar results were obtained by Arusoğlu et al. [17], showing that individuals with higher OCD symptoms had greater ON tendencies (Table 1).

**Table 1:** Cronbach’s alpha related to each subscale used in the present study

EHQ_Total score	Polish sample	0.92
	Italian sample	0.90
EHQ_Knowledge	Polish sample	0.83
	Italian sample	0.83
EHQ_Problems	Polish sample	0.88
	Italian sample	0.85
EHQ_Feelings	Polish sample	0.80
	Italian sample	0.70
OCI_R_Total score	Polish sample	0.92
	Italian sample	0.86
OCI_R_Hoarding	Polish sample	0.71
	Italian sample	0.79
OCI_R_Ordering	Polish sample	0.75
	Italian sample	0.85
OCI_R_Mental Neutralizing	Polish sample	0.77
	Italian sample	0.84
OCI_R_Washing	Polish sample	0.71
	Italian sample	0.75
OCI_R_Obsessing	Polish sample	0.84
	Italian sample	0.89
OCI_R_Checking	Polish sample	0.82
	Italian sample	0.74
OBQ_Total score	Polish sample	0.96
	Italian sample	0.97
OBQ_RT	Polish sample	0.91
	Italian sample	0.89
OBQ_PC	Polish sample	0.93
	Italian sample	0.88
OBQ_ICT	Polish sample	0.90
	Italian sample	0.91
MPS_Total score	Polish sample	0.92
	Italian sample	0.91
MPS_PS	Polish sample	0.85
	Italian sample	0.85
MPS_O	Polish sample	0.89
	Italian sample	0.89
MPS_CMD	Polish sample	0.92
	Italian sample	0.91
MPS_PEPC	Polish sample	0.85
	Italian sample	0.87

**Fig. 1:**
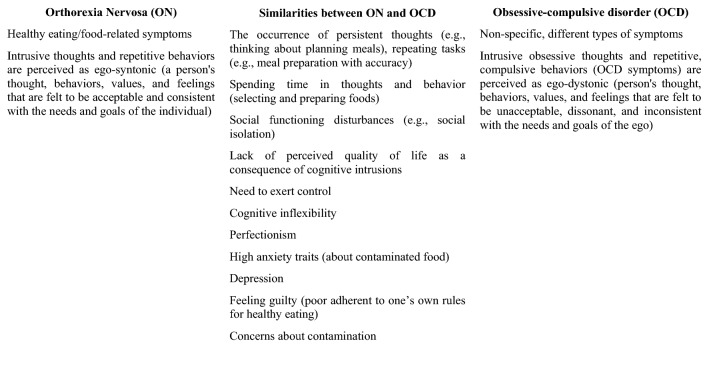
Orthorexia Nervosa (ON) and obsessive–compulsive disorder (OCD): characteristics and similarities (based on 5, 9, 11, 20)

On the other hand, some studies have shown that ON prevalence rates among OCD patients are low [18]. Nevertheless, there is initial evidence that ON and OCD exhibit similar behavioral and thinking patterns (e.g., [5, 9, 11, 19]. Analysis of ON reveals several overlapping characteristics with OCD (see Fig. 1).

### Relationship between Orthorexia Nervosa and Obsessive–Compulsive Disorder

The researchers’ point of view on ON’s nature turns out to be inconsistent with each other. On the one hand, previous studies have related ON with OCD symptoms in healthy samples [16, 17, 21–23]. Hayes et al. [23] found that ON symptoms were associated with OCD symptoms, perfectionism, disordered eating, and appearance anxiety among undergraduate students. Bundros et al. [22] found that higher ON tendencies were associated with increased obsessive–compulsive and body dysmorphic traits among college students.

### Orthorexia Nervosa and other disorders: the overlapping features

Beyond OCD and AN, ON shares overlapping features with several other diagnostic categories, including obsessive–compulsive personality disorder (OCPD). Similarities between the two conditions include perfectionism, rigid thinking, preoccupation with details and perceived rules, hyper morality, and excessive devotion [11]. Moreover, ON can be considered a combination of AN and OCD because it shares several characteristics of both disorders [21]. Neurocognitive deficits, including impairments in set-shifting (flexible problem solving), external attention, and working memory, have been identified in patients with ON, AN, and OCD [11, 22]. Possible biochemical similarities have been found that can influence thought patterns and behaviors in both ON and OCD [21]. Patients with OCD and eating disorders have higher cerebral glucose metabolism, which does not allow them to effectively complete a task that required the work of the prefrontal cortex and caudate nucleus of the brain [23]. Murphy et al. [23] implied that similar neurophysiological similarities exist in OCD and ON. 

### Cross-cultural comparison of Orthorexia Nervosa

There is limited evidence of a link between ON and OCD, and no definitive conclusions can be drawn. The interplay between socio-cultural context and ON has been poorly investigated as well. In the literature, the cross-cultural comparison has been described as comparisons by which established practices and behaviors from two or more different countries or societies are contrasted and reflected against the respective countries’ cultural particularities [26]. Previous cross-cultural studies have suggested that populations that prioritize pleasure over health, e.g., southern European populations, demonstrate healthier eating behaviors [27]. One might expect that the Mediterranean diet and the convivial and social value attributed to eating [20] may play an important role in the Italian sample and be less associated with ON than in the Polish sample. In Poland’s last years, good consumer practices have gained greater popularity, and healthy eating has become the most important among the strategies aimed at improving one’s state of health [28], which may engender an increased preoccupation regarding restrictive dietary practices believed by the individuals to promote optimum health. The previous research showed that a higher prevalence of ON was found among the Polish samples than Italian and Spanish one [20].

The objectives of the present study were: (1) to examine the differences in ON and OCD symptoms among university students in Poland and Italy and (2) to investigate the relationship between ON and OCD symptoms among Polish and Italian university students; (2). Based on the previous research [20, 29], we hypothesize that:

H1: Polish students show more ON behaviors than Italian ones.

H2: Students with a high level of ON present high levels of OCD symptoms.

H3: ON is related to OCD symptoms in both Italian and Polish students.

## Methods

### Participants and procedure

The study group consisted of 286 (47.2%) Polish and 320 (52.8%) Italian psychology students, respectively, recruited from different universities located in Poland (e.g., Silesian region, Upper Silesia region) and Italy (e.g., northern Italy).

In both countries, recruitment took place during university lessons; specifically, students were given a short general presentation of the project and proposed participating in the research. Individuals had to confirm their participation via email. Through creating an individual account managed and monitored by themselves, the authors sent participants an email with the link to access the protocol through Google Forms. Participants responded to the questionnaires in a single online session; first, they had to sign the informed consent form and fill in the personal datasheet. Then, a battery of counterbalanced self-report questionnaires was administered. A numerical code corresponding accompanied the informed consent form to the one reported on the tests and the personal datasheet. No one of the students was excluded from the research. Protocols with at least 10% of the answers omitted have been excluded. The questionnaires were administered based on an electronic format by a properly trained psychologist.

A research ethics committee has approved the present study (no. WKEB59/05/2019 in Poland and no. 3067—14/06/2019 in Italy). All procedures performed in our study were following the 1964 Helsinki declaration (adopted by the 18th World Medical Association General Assembly, Helsinki, Finland) and its later amendments or comparable ethical standards.

### Measures

#### Demographic schedule

Participants were asked to complete a schedule to collect demographics data (e.g., gender, age, years of education, Body Mass Index), with also other information regarding what kind of diet they were on, if/why they were avoiding particular food and if they had another medical or psychological diagnosis.

#### The Eating Habits Questionnaire (EHQ)

The EHQ [30–32] is a 21-item self-report questionnaire aimed to assess ON symptoms on a four-point Likert scale (“false, not at all true,” “slightly true,” “mainly true,” and “very true”). It is characterized by three subscales named “Knowledge” (refers to knowledge about healthy eating), “Feelings” (it concerns feelings, emotions, and sensations related to conducting a healthy diet), and "Problems". Based on the initial validation [30], a 3-factor model fit was examined with confirmatory factor analysis. The internal consistency (0.82 < Cronbach’s *α* < 0.90) and test–retest reliability (0.72 < r < 0.81) of the subscales remained good once 14 poorly fitting items were deleted.

The Italian validation [31], both exploratory and confirmatory factorial analysis, evidence the same original EHQ structure [30]. Moreover, a good internal consistency and a one-month test–retest reliability were highlighted (r ranging from 0.50 to 0.75; 0.001 < *p* < 0.01). Both the original and Italian versions highlighted adequate internal consistency indices and convergent and divergent validity [30, 31]. Regarding the Polish validation of the EHQ [32], the three-factor structure showed satisfactory goodness-of-fit (comparative fit index (CFI) = 0.99, root mean square error of approximation (RMSEA) = 0.008). Reliability analysis for the Polish version of the EHQ across the whole questionnaire showed strong internal consistency (*α* = 0.88, intraclass correlation coefficient (ICC) = 0.86).

This study showed Italian and Polish samples’ adequate reliability (for total and subscales scores, Cronbach’s *α* ranging from 0.70 and 0.92).

#### The Obsessive–Compulsive Inventory-Revised (OCI-R)

The OCI-R [33–35] is an 18-item self-report questionnaire assessing the Obsessive–Compulsive Disorder (OCD) symptoms on a five-point Likert scale (“Not at all”, “A little”, “Moderately”, “A lot” and “Extremely”). The questionnaire is composed of six subscales (“Washing”, “Ordering”, “Hoarding”, “Mental Neutralizing”, “Obsessing”, and “Checking”) composing an additional final total score. The original version has put in evidence good reliability and validity indices of the OCI-R, showing strong convergence with established measures of OCD, moderate to high internal consistency across the six subscales, and adequate to high test–retest stability [33]. As regards the Italian version [34], the confirmatory factor analysis showed the original six factors structure. Moreover, a good internal consistency is confirmed (0.76 < Cronbach's *α* < 0.94). A 30-day test–retest reliability was good (0.76 < r < 0.99) and convergent, discriminant and criterion validity were acceptable [34]. As regards the Polish version [35], the results showed adequate test reliability for the full-scale and subscales scores, high internal consistency (0.62 < Cronbach's *α* < 0.85), and confirmed satisfactory convergent and divergent validity [36]. In this study, the Italian and Polish self-report questionnaire’s good internal consistency is highlighted both for the total and the subscales scores (Cronbach’s *α* ranging from 0.71 and 0.92).

#### The Multidimensional Perfectionism Scale (MPS)

The MPS [37–40] is a 35-item self-report questionnaire used to assess perfectionism features on a five-point Likert scale (from “Strongly agree” to “Strongly Disagree”). The MPS is composed of six subscales: “Concern over Mistakes”, “Personal Standards”, “Parental Expectations”, “Parental Criticism", "Doubts about actions", and "Organization". The internal consistency was good (0.77 < Cronbach's *α* < 0.93) [37].

Regarding the Italian version [39], the confirmatory factor analysis confirmed the original structure. Moreover, the internal consistency was good (0.76 < Cronbach's *α* < 0.87), and the concurrent validity was acceptable. Regarding the Polish version, the adapted version of the MPS most appropriate factor structure contains five correlated factors without the “Organization” subscale. Reliability (Cronbach’s *α*) of the MPS dimensions ranged from 0.70 to 0.91 [40]. Cronbach’s alpha in this study was adequate for the Italian and Polish samples' total and subscales scores (Cronbach's *α* ranging from 0.85 and 0.92).

#### The Obsessive Beliefs Questionnaire (OBQ)

The OBQ [41–44] is a self-report questionnaire designed to investigate the core cognitive domains in the origin and maintenance of OCD. The initial 87-item version of the scale (OBQ-87) is composed of six subscales (OCCWG, 2001) highly inter-correlated; for this reason, the OCCWG’s [43] empirically derived the OBQ-44 version. The OBQ-44 is composed of six subscales: “Responsibility”, “Certainty”, “Perfectionism”, “Threat estimation", "Control of thoughts", and "Importance of thoughts". Regarding the Polish translation, the OBQ was translated from English to Polish using a standard forward–backward translation procedure. The English version of the OBQ was first translated into Polish (by two translators who independently translated the same questionnaire) and then back-translated into English (by two independent native English speakers without reference to the English original).

Based on the OBQ-87, an Italian 44-item version [44] has been derived for this study to compare Italian and Polish samples. In this study, all subscales demonstrated good internal consistency (0.88 < Cronbach's *α* < 0.97) both in the original and derivate versions.

## Data analysis

All analyses were carried out using the SPSS Statistics-Version 22. First, Cronbach’s alpha was investigated for all the administered self-report questionnaires.

Multivariate ANOVA, and ANCOVA, and a Chi-squared index were used to explore the differences between groups. Considering the Polish sample’s hypothesis had major scores, we have set the alpha level at 0.05 for a one-tailed test. Correlation and partial correlation analysis were used to investigate the relationship between the EHQ and other questionnaires. Finally, Pearson’s r correlations have been done to investigate relations between ON and the other constructs.

## Results

### Demographic features of the Polish and Italian university students’ samples

Demographic characteristics of study population are presented in Table 2.

**Table 2:** The main demographic features of the Polish and Italian university students’ samples

	Country	M (SD) or N (%)	*F* or Chi-squared	*p*	Partial *η* 2
Age	Polish sample	22.33 (2.38)	3.67	0.06	0.006
	Italian sample	21.98 (2.09)			
BMI	Polish sample	22.69 (4.33)	17.28	< 0.001	0.03
	Italian sample	21.43 (3.07)			
Gender (% female)	Polish sample	236 (82.5%)	0.79	0.38	0.04
	Italian sample	255 (79.7%)			
Marital status (% single)	Polish sample	266 (93%)	1.42	0.23	0.05
	Italian sample	289 (90.3%)			
Chronic disease	Polish sample	53 (18.5%)	14.43	< 0.001	0.15
	Italian sample	26 (8.1%)			
Mood disorder	Polish sample	24 (8.4%)	13.63	< 0.001	0.15
	Italian sample	6 (1.9%)			
Anxiety disorder	Polish sample	21 (7.3%)	1.06	0.30	0.04
	Italian sample	17 (5.3%)			
Avoidance of food	Polish sample	103 (36.7%)	3.29	0.07	0.07
	Italian sample	95 (29.7%)			
Using laxatives	Polish sample	11 (3.9%)	3.19	0.07	0.07
	Italian sample	5 (1.6%)			
Vomiting	Polish sample	10 (3.6%)	1.02	0.31	0.04
	Italian sample	7 (2.2%)			
Diet type (omnivores)	Polish sample	188 (66.4%)	76.18 < 0.001 0.36		
	Italian sample	297 (92.8%)			
Diet type (fruitarians/vegetarians/vegans)	Polish sample	28 (9.9%)			
	Italian sample	17 (5.3%)			

As shown in Table 2, no statistically significant differences emerged for age, gender, and marital status between the Polish and Italian samples. Otherwise, a difference between groups regarding the BMI has been put in evidence; specifically, Polish university students have, on average, a higher Body Mass Index. Polish sample includes a greater number of individuals with chronic disease and a Mood disorder; instead, no differences emerged for the presence of an Anxiety disorder. No differences were put in evidence in the avoidance of food, in using laxatives, and in vomiting. Differences between groups also emerged for the diet type, with the Italian sample composed of a greater number of omnivores, rather than the Polish one, and the Polish group composed of a greater number of fruitarians/vegetarians/vegans.

### Invariance’s measurement across groups

The invariance models could be considered adequate. Indeed, although the last analysis concerning the mean invariance is not satisfied, the estimation could be regarded as acceptable.

### Comparison between Polish and Italian university students in ON, OCD symptoms, core beliefs of OCD, and perfectionism features

ON, OCD symptoms, core beliefs of OCD, and perfectionism features among university students are presented in Table 3.

**Table 3:** DWLS estimation method

Model	*χ*^2^	df	*χ*^2^/df	RMSEA	90% RMSEA	SRMR	CFI	Comparison	Delta *χ*^2^	Delta df	*p*-value	Delta CFI	Decision
CI	930,375	372	2,501	0,071	(0.066–0.077)	0,096	0,979						
LI	1060,246	390	2,719	0,076	(0.071–0.082)	0,103	0,975	Model CI vs LI	129,871	18	0,000	− 0.004	Accepted
TI	1145,522	429	2,670	0,075	(0.070–0.081)	0,098	0,973	Model TI vs LI	85,276	39	0,000	− 0.002	Accepted
FVI	1176,027	432	2,722	0,076	(0.071–0.082)	0,099	0,972	Model FVI vs TI	30,505	3	0,000	− 0.001	Accepted
FCI	1236,657	435	2,843	0,079	(0.074–0.084)	0,101	0,970	Model FCI vs FVI	60,630	3	0,000	− 0.002	Accepted
FMI	1443,375	438	3,295	0,088	(0.083–0.093)	0,102	0,962	Model FMI vs FCI	206,718	3	0,000	− 0.008	Reject

*CI* Full Configural Invariance, *LI* Full Factor Loadings Invariance, *TI* Full Thresholds Invariance, *FVI* Full Factor Variance Invariance, *FCI* Full Factor Covariance Invariance, *FMI* Full Factor Mean Invariance

Polish sample had, on average, higher scores than the Italian one in the EHQ-21 (total score, "Problems" and "Feelings" scales), the OCI-R (total score, "Hoarding", "Ordering", "Mental Neutralizing", "Washing” and “Checking”), the OBQ (total score, “Responsibility/Threat estimation” and “Perfectionism/Certainty” scales), and the MPS (“Organization” and “Parental expectations and criticism”) (4,18 < *F* < 29,86; *p* < 0.05).

Instead, the Italian sample had higher scores than the other one in the OCI-R "Obsessing", the OBQ "Importance/Control of Thoughts”, and the MPS “Concern over Mistakes/Doubting of Action” (3,13 < *F* < 17,41; *p* < 0.05).

### Assessment of high versus low level of ON with OCD symptoms and core beliefs of OCD and perfectionism features

Two groups of individuals (named High-EHQ and Low-EHQ, respectively) have been set up: the Italian and the Polish sample separately have been divided according to the EHQ cut-off score. Specifically, the High-EHQ subjects obtained a score equal to or greater than the 90 percentile (Polish group's raw score >  = 51; Italian group's raw score >  = 45), while the Low-EHQ individuals were casually extracted from the group with a score less than the 50 percentile (Polish group's raw score < 33; Italian group's raw score < 32).

A multivariate ANOVA and ANCOVA have been performed considering the two factors “Country” and “Low/High EHQ” (Table 4) and, as dependent variables, the OCI-R, the MPS, and the OBQ total and subscales scores have been considered. Moreover, the BMI effect has been controlled. Significant differences emerged for the single factors "Country" (*F* = 8.59; *p* < 0.001; partial *η*^2^ = 0.60), for the “High/Low EHQ” (*F* = 7.34; *p* < 0.001; partial *η*^2^ = 0.56) and the interaction (*F* = 9.24; *p* < 0.001; partial *η*^2^ = 0.61).

**Table 4:** Comparison between the Polish and Italian samples controlling for the BMI effect

	Country	M (SD)	*F*	*p*	Partial *η*^2^
EHQ_Total score	Polish sample	34.95 (10.54)	4.18	< 0.05	0.02
	Italian sample	33.25 (8.57)			
EHQ_Knowledge	Polish sample	9.46 (3.42)	2.44	ns	0.01
	Italian sample	10.12 (3.53)			
EHQ_Problems	Polish sample	16.39 (5.56)	9.46	< 0.001	0.03
	Italian sample	14.99 (4.24)			
EHQ_Feelings	Polish sample	9.10 (3.22)	9.93	< 0.001	0.04
	Italian sample	8.14 (2.55)			
OCI_R_Total score	Polish sample	17.81 (13.95)	12.86	< 0.001	0.05
	Italian sample	13.09 (8.66)			
OCI_R_Hoarding	Polish sample	3.64 (3.00)	6.86	< 0.01	0.02
	Italian sample	2.80 (2.35)			
OCI_R_Ordering	Polish sample	3.62 (3.02)	3.62	< 0.05	0.01
	Italian sample	2.99 (2.53)			
OCI_R_Mental Neutralizing	Polish sample	1.44 (2.36)	18.63	< 0.001	0.06
	Italian sample	0.63 (1.35)			
OCI_R_Washing	Polish sample	2.73 (2.76)	26.05	< 0.001	0.09
	Italian sample	1.38 (1.92)			
OCI_R_Obsessing	Polish sample	2.47 (2.92)	3.13	< 0.05	0.01
	Italian sample	3.11 (2.97)			
OCI_R_Checking	Polish sample	3.90 (3.39)	26.24	< 0.001	0.09
	Italian sample	2.19 (2.18)			
OBQ_Total score	Polish sample	141.95 (47.56)	5.97	< 0.01	0.02
	Italian sample	129.69 (40.62)			
OBQ_RT	Polish sample	53.23 (18.09)	20.60	< 0.001	0.07
	Italian sample	44.40 (15.19)			
OBQ_PC	Polish sample	57.13 (20.13)	12.52	< 0.001	0.04
	Italian sample	49.56 (15.64)			
OBQ_ICT	Polish sample	31.59 (13.80)	6.82	< 0.01	0.02
	Italian sample	35.73 (13.66)			
MPS_Total score	Polish sample	100.20 (22.26)	0.26	ns	0.001
	Italian sample	98.95 (19.91)			
MPS_PS	Polish sample	22.43 (6.13)	1.13	ns	0.004
	Italian sample	21.69 (5.50)			
MPS_O	Polish sample	23.20 (4.98)	5.80	< 0.01	0.02
	Italian sample	21.93 (5.15)			
MPS_CMD	Polish sample	33.21 (11.89)	17.41	< 0.001	0.06
	Italian sample	38.97 (10.94)			
MPS_PEPC	Polish sample	21.36 (8.44)	29.86	< 0.001	0.10
	Italian sample	16.36 (6.91)			

*EHQ* Eating Habits Questionnaire, *OCI-R* Obsessive Compulsive Inventory-Revised, *OBQ* Obsessive Beliefs Questionnaire, *OBQ_RT* Obsessive Beliefs Questionnaire – Responsibility/Threat estimation, *OBQ_PC* Obsessive Beliefs Questionnaire – Perfectionism/Certainty, *OBQ_ICT* Obsessive Beliefs Questionnaire – Importance/Control of Thoughts, *MPS* Multidimensional Perfectionism Scale, *MPS_PS* Multidimensional Perfectionism Scale – Personal Standard, *MPS_O* Multidimensional Perfectionism Scale – Organization, *MPS_CMD* Multidimensional Perfectionism Scale – Concern over Mistakes/Doubting of Action, *MPS_PEPC*  Multidimensional Perfectionism Scale – Parental Expectations/Parental Criticism

Specifically, the Polish High-EHQ group, rather than the Italian one, obtained greater scores in the OCI-R total score, the “Hoarding” subscale, the “Mental Neutralization” scale, the "Washing" scale, and the MPS "Parental Expectations/Parental Criticism” scale (2.52 < *F* < 9.29; *p* < 0.09; 0.003 < partial *η*^2^ < 0.26). Otherwise, the Italian High-EHQ group obtained greater scores in the MPS “Organization” and the “Concern over Mistakes/Doubting of Action” scales (3.56 < *F* < 6.64; *p* < 0.05; 0.12 < partial *η*^2^ < 0.20).

Polish Low-EHQ group, rather than the Italian one, obtained greater scores in the OCI-R total score, the “Ordering” subscale, the OBQ total score, the “Perfectionism/Certainty” scale, the MPS total, the “Concern over Mistakes/Doubting of Action”, and the “Parental Expectations/Parental Criticism” scales (9.72 < *F* < 104.61; *p* < 0.001; 0.02 < partial *η*^2^ < 0.79).

Otherwise, the Italian Low-EHQ group, rather than the Polish one, obtained greater scores in the OCI-R “Checking” scale, the “OBQ “Importance/Control of Thoughts" scale, and the MPS "Organization" scale (12.79 < *F* < 193.23; *p* < 0.001; 0.32 < partial *η*^2^ < 0.88).

### Examination of the relationship between ON, OCD symptoms, core beliefs of OCD, and perfectionism features among Polish and Italian university students

The associations between ON and investigated variables are shown separately for Polish and Italian university students (Table 5).

**Table 5:** Assessment of high versus low level of ON with OCD symptoms and core beliefs of OCD and perfectionism features: comparison between Polish and Italian samples

	Country (Polish: *N* = 49; Italian: *N* = 64)	Low/High EHQ (Low = 58; High = 55)	*M* (SD)
OCI_R Total score	Polish sample	Low	16.88 (11.55) 23.46 (17.19) 20.10 (14.81)
		High	
		Total	
	Italian sample	Low	2.88 (3.12) 17.32 (11.45) 9.88 (10.97)
		High	
		Total	
	Total	Low	8.91 (10.52) 20 (14.43)
		High	
OCI_R_Hoarding	Polish sample	Low	3.60 (2.97) 4.58 (2.93) 4.08 (2.96)
		High	
		Total	
	Italian sample	Low	2.58 (2.68) 2.74 (2.31) 2.66 (2.49)
		High	
		Total	
	Total	Low	3.02 (2.83) 3.55 (2.73)
		High	
OCI_R_Ordering	Polish sample	Low	3.04 (2.3) 4.33 (3.20) 3.67 (2.82)
		High	
		Total	
	Italian sample	Low	0.52 (0.97) 3.87 (2.71) 2.14 (2.61)
		High	
		Total	
	Total	Low	1.60 (2.09) 4.07 (2.91)
		High	
OCI_R_Mental Neutralizing	Polish sample	Low	0.92 (1.35) 2.92 (3.72) 1.90 (2.92)
		High	
		Total	
	Italian sample	Low	1.52 (2.06) 1.03 (1.80) 1.28 (1.94)
		High	
		Total	
	Total	Low	1.26 (1.80) 1.85 (2.93)
		High	
OCI_R_Washing	Polish sample	Low	2.68 (2.69) 4.04 (3.58) 3.35 (3.20)
		High	
		Total	
	Italian sample	Low	2.39 (2.21) 2.16 (2.93) 2.28 (2.57)
		High	
		Total	
	Total	Low	2.52 (2.41) 2.98 (3.34)
		High	
OCI_R_Obsessing	Polish sample	Low	2.40 (2.48) 3.58 (3.32) 2.98 (2.95)
		High	
		Total	
	Italian sample	Low	1.91 (2.43) 4.74 (3.92) 3.28 (3.52)
		High	
		Total	
	Total	Low	2.12 (2.44) 4.24 (3.69)
		High	
OCI_R_Checking	Polish sample	Low	4.24 (3.42) 4 (3.44) 4.12 (3.40)
		High	
		Total	
	Italian sample	Low	24.94 (5.24) 2.77 (2.99) 14.20 (11.95)
		High	
		Total	
	Total	Low	16.02 (11.28) 3.31 (3.22)
		High	
OBQ_Total score	Polish sample	Low	140.72 (50.51) 145.92 (45.50) 143.27 (47.69)
		High	
		Total	
	Italian sample	Low	93.55 (21.87) 148 (48.05) 119.92 (45.77)
		High	
		Total	
	Total	Low	113.88 (43.57) 147.09 (46.53)
		High	
OBQ_RT	Polish sample	Low	52.32 (20.08) 54.88 (17.69) 53.57 (18.79)
		High	
		Total	
	Italian sample	Low	46.18 (16.05) 49.69 (18.13) 47.88 (17.04)
		High	
		Total	
	Total	Low	48.83 (17.99) 51.95 (17.96)
		High	
OBQ_PC	Polish sample	Low	57.28 (22.20) 55.92 (18.12) 56.61 (20.10)
		High	
		Total	
	Italian sample	Low	36.30 (13.58) 56.84 (16.34) 46.25 (18.10)
		High	
		Total	
	Total	Low	45.34 (20.51) 56.44 (16.98)
		High	
OBQ_ICT	Polish sample	Low	31.12 (12.85) 35.13 (13.75) 33.08 (13.31)
		High	
		Total	
	Italian sample	Low	121.61 (42) 41.47 (17.92) 82.79 (51.75)
		High	
		Total	
	Total	Low	82.60 (55.71) 38.70 (16.40)
		High	
MPS_Total score	Polish sample	Low	98.08 (26.91) 104.33 (19.82) 101.14 (23.67)
		High	
		Total	
	Italian sample	Low	23 (14.75) 110.39 (19.00) 65.33 (47.12)
		High	
		Total	
	Total	Low	55.36 (42.82) 107.75 (19.42)
		High	
MPS_PS	Polish sample	Low	21.52 (7.00) 24.13 (5.69) 22.80 (6.46)
		High	
		Total	
	Italian sample	Low	21.21 (5.30) 23.65 (4.89) 22.39 (5.21)
		High	
		Total	
	Total	Low	21.34 (6.04) 23.85 (5.21)
		High	
MPS_O	Polish sample	Low	22.88 (5.73) 21.83 (5.53) 22.37 (5.60)
		High	
		Total	
	Italian sample	Low	35.09 (11.36) 24.84 (3.49) 30.13 (9.90)
		High	
		Total	
	Total	Low	29.83 (11.11) 23.53 (4.70)
		High	
MPS_CMD	Polish sample	Low	32.04 (13.76) 35.04 (11.20) 33.51 (12.53)
		High	
		Total	
	Italian sample	Low	14.91 (7.21) 43.13 (10.94) 28.58 (16.89)
		High	
		Total	
	Total	Low	22.29 (13.50) 39.60 (11.67)
		High	
MPS_PEPC	Polish sample	Low	21.64 (7.51) 23.33 (9.68) 22.47 (8.59)
		High	
		Total	
	Italian sample	Low	3.03 (3.74) 18.77 (8.01) 10.66 (10.03)
		High	
		Total	
	Total	Low	11.05 (10.86) 20.76 (8.99)
		High	

OCI-R = Obsessive Compulsive Inventory-Revised; OBQ = Obsessive Beliefs Questionnaire; OBQ_RT = Obsessive Beliefs Questionnaire – Responsibility/Threat estimation; OBQ_PC = Obsessive Beliefs Questionnaire – Perfectionism/Certainty; OBQ_ICT = Obsessive Beliefs Questionnaire – Importance/Control of Thoughts; MPS = Multidimensional Perfectionism Scale; MPS_PS = Multidimensional Perfectionism Scale – Personal Standard; MPS_O = Multidimensional Perfectionism Scale—Organization; MPS_CMD = Multidimensional Perfectionism Scale – Concern over Mistakes/Doubting of Action; MPS_PEPC = Multidimensional Perfectionism Scale – Parental Expectations/Parental Criticism

In general, correlations are low (0.13 < *r* < 0.28; *p* < 0.05) as confirmation of partial independence from OCD symptoms and beliefs in both Polish and Italian university students (Table 6).

**Table 6:** Correlations considering Polish and Italian samples

	R	EHQ Total score	EHQ Knowledge	EHQ Problems	EHQ Feelings
OCI_R_total score	Polish/Italian	0.13*/0.22***	0.02/0.11	0.17*/0.23***	0.10/0.22**
OCI_R_Hoarding	Polish/Italian	0.13*/0.05	0.03/− 0.02	0.14*/0.03	0.14*/0.14*
OCI_R_Ordering	Polish/Italian	0.10/0.18**	0.01/0.15*	0.12/0.18**	0.11/0.10
OCI_R_MN	Polish/Italian	0.18**/0.11	0.08/− 0.01	0.25**/0.17**	0.06/0.09
OCI_R_Washing	Polish/Italian	0.13**/0.19**	0.03/0.13*	0.21**/0.17**	0.03/0.19**
OCI_R_Obsessing	Polish/Italian	0.08/0.19**	− 0.02/0.09	0.13*/0.19**	0.05/0.20**
OCI_R_Checking	Polish/Italian	0.03/0.13*	− 0.02/0.05	0.03/0.16**	0.08/0.12*
OBQ_TOT score	Polish/Italian	0.03/0.17**	− 0.03/0.05	0.05/0.17**	0.04/0.23***
OBQ_RT	Polish/Italian	0.05/0.12*	0.01/0.01	0.04/0.11*	0.07/0.18**
OBQ_PC	Polish/Italian	− 0.03/0.19**	− 0.06/0.11	− 0.02/0.17**	0.01/0.22***
OBQ_ICT	Polish/Italian	0.08/0.15*	− 0.01/0.01	0.14*/0.17**	0.03/0.21***
MPS_TOT	Polish/Italian	0.12/0.28***	0.05/0.18**	0.15*/0.27***	0.10/0.26***
MPS_PS	Polish/Italian	0.15*/21**	0.15*/0.19***	0.12/0.15**	0.13/0.18*
MPS_O	Polish/Italian	− 0.01/0.18***	0.05/0.22***	− 0.08/0.14*	0.07/0.07
MPS_CMD	Polish/Italian	0.09/0.21***	− 0.03/0.11	0.15*/0.21***	0.05/0.22***
MPS_PEPC	Polish/Italian	0.09/0.18***	0.03/0.04	0.13*/0.21***	0.05/0.20***

*EHQ* Eating Habits Questionnaire, *OCI-R*  Obsessive Compulsive Inventory-Revised, *OBQ*  Obsessive Beliefs Questionnaire, *OBQ_RT*  Obsessive Beliefs Questionnaire – Responsibility/Threat estimation, *OBQ_PC*  Obsessive Beliefs Questionnaire – Perfectionism/Certainty, *OBQ_ICT* Obsessive Beliefs Questionnaire – Importance/Control of Thoughts, *MPS*  Multidimensional Perfectionism Scale, *MPS_PS* Multidimensional Perfectionism Scale – Personal Standard, *MPS_O* Multidimensional Perfectionism Scale – Organization, *MPS_CMD* Multidimensional Perfectionism Scale – Concern over Mistakes/Doubting of Action, *MPS_ PEPC* Multidimensional Perfectionism Scale – Parental Expectations/Parental Criticism

*p < 0.05; **p < 0.01 ***p < 0.001

## Discussion

This study had two main objectives: to examine the differences in ON and OCD symptoms among Polish and Italian university students and to investigate the relationship between ON and OCD symptoms among two samples of university students.

Our findings showed that Polish students have more obsessive and perfectionistic traits and greater feelings and ON’s features than Italians. Moreover, for the high and low EHQ groups’ multivariate analysis, differences between Polish and Italian emerged and the interaction between ON and culture factors, regardless of the BMI. Polish individuals showed higher levels of OCD symptoms (excepted for the Obsessing and Checking scales), core beliefs (excepted for the “ICT" scale), and perfectionistic traits (excepted for the "Organization" scale). To understand in more detail the impact of the Country factor, we have also compared, respectively, Polish and Italian students with higher ON scores and Polish and Italian ones with lower scores: Polish students, both with lower and higher ON, shown more obsessive and compulsive features than the other groups and, for all these reasons, we could affirm that the first hypothesis was confirmed.

In line with these results finding that Polish students displayed higher levels of orthorexic eating behavior than Italian students, recent studies have shown higher ON behaviors in the Polish sample than Italian ones (82% vs. 46% [29]; 66.5% vs. 30.9% [31]). The Mediterranean diet, dominant in Italy, has long been considered one of the world’s healthiest and to have been linked with several health benefits [28]. In contrast, in Poland, over the past decades, economic and political changes have influenced the lifestyle-related behaviors of various social groups, especially on young people [28], and a new trend seems to focus on the health aspects of food choices [45]. Control of the composition of food products and the belief that healthy dieting is the most important approach for improving one’s health could explain the higher levels of ON found in the Polish sample. On the other hand, likely, that the lowest frequency of ON and attitudes found in the Italian sample depends on an enogastronomic culture that includes the Mediterranean diet style [20].

The preset study’s second objective was to evaluate the ON level (high versus low) with OCD symptoms and core beliefs of OCD and perfectionism. Our results demonstrated that students having a higher level of ON exhibited higher levels of OCD symptoms (excepted for the Hoarding, the Mental Neutralizing, and the Washing scales), OCD core beliefs (excepted for the "Responsibility/Threat estimation" scale), and perfectionism (excepted for the "Personal Standard" scale) (6.91 < *F* < 201.21; *p* < 0.01; partial *η*^2^ < 0.79). Based on these data, the second hypothesis was confirmed. Our findings concur with other studies showing that participants with ON features had a significantly higher level of OCD symptoms and beliefs [27]. Obsessive and compulsive behaviors were more pronounced in ON, and more than about 30% of ON subjects fulfilled criteria for clinically relevant OCD symptoms (compared to 11.2% in non-ON) [14].

Based on our knowledge, this is the first study that analyzed this relation, and for this reason, future studies have been realized for further investigation.

The third objective of the present study was to investigate the relationship between ON and OCD symptoms, core beliefs of OCD, and perfectionism among Polish and Italian university students. Our results showed that, in general, correlations were low (− 0.08 < *r* < 0.12; *p* < 0.05) as confirmation of partial independence ON from OCD symptoms and core beliefs of OCD in both Polish and Italian university students (H3 was partially confirmed). These results suggest that obsessive symptomatology could be considered an ON epiphenomenon. Moreover, ON seems similar to OCD for repetitively, intrusively, and rigidity behaviors and consequences (e.g., wasting time). Our findings are consistent with other studies revealing that greater ON symptomatology is associated with greater levels of OCD symptoms [17, 22, 24, 25, 30, 46], confirming that an association does exist and may mean that ON symptoms and OCD symptoms are comorbid. The longitudinal study could answer the question of whether ON tendencies, at some point in time, may prompt a person to develop routine, repetitive behaviors [21] as well as to develop “pure” diets [46] and may ultimately lead to OCD like-behavior.

In the previous study [46], checking and dressing/grooming compulsions showed ON’s most significant correlations, suggesting parallel cognitive processes with the ritualistic compulsions. The dressing/grooming compulsions represent the urge to insist on doing hygiene steps in a fixed sequence. If the sequence is interrupted, patients may again start initially, which suggests a phenomenological similarity with the ritualistic behavior of preparing food in people with ON [46]. Our results contrast to previous findings: first, no relationships between ON and OCD (except for checking) have been shown. These data confirm the hypothesis that ON and OCD are two different clinical syndromes [47] and show that knowledge of healthy eating has slight negative correlations with OCD symptoms and problems related to ON moderately correlated with OCD symptoms [48].

We found weak and moderate associations between ON and perfectionism features, especially considering the Italian sample. Recent studies have also demonstrated that perfectionism was positively correlated with greater ON symptomatology among university students [19, 25, 49]. Besides, all components of perfectionism were significantly correlated and predicted ON problems. Only the tendency of individuals to set excessively high standards for themselves ("personal standards and organization") was significantly correlated with behaviors and feelings related to ON [49]. Perfectionism, setting high expectations and standards, worrying, fear, or doubtfulness about the future, being emotionally restrained, or demonstrating intrusive feelings/thoughts and repetitive behavior could be determinants of orthorexic behaviors [8].

The current study has limitations. The first is sampling bias — our sample (university students) does not reflect the general population’s characteristics. In addition, the selection of university students was not randomized. Self-reported data cannot be independently verified and may lead to inaccuracies (e.g., social desirability bias). A cross-sectional study design does not evaluate causality due to the non-temporal nature of the study design. Online questionnaires could have decreased participant recruitment’s internal validity. Students’ awareness in participating in the study can also influence outcomes (Hawthorne effect) [50]. Finally, the non-validated version of the OBQ based on the Polish population should be considered a limitation of the present study.

## Conclusion

The present results demonstrated that ON is related to OCD symptoms and perfectionism features. Further investigations regarding the correlates of ON across different cultural groups are still needed. Moreover, it is important to identify whether ON is similar or different from other disorders (e.g., AN and OCD). Moreover, further studies should include three clinical subgroups, respectively, diagnosed with ON, AN, and OCD, so this could resolve the debate on the nature of ON and its treatment. Our findings may suggest screening for OCD symptomology as a comorbid problem among individuals with ON.

Longitudinal studies are also needed to assess the ON develops; if it develops independently, it is a prelude or a result of AN recovery or a variant of the OCD spectrum. More studies are needed comparing those who only have ON and individuals with OCD to evaluate how psychological and clinical profiles are similar amongst the ON and OCD.

## What is already known on this subject?

Based on the literature, ON shares several characteristics with other psychological disorders, including Obsessive–Compulsive Disorder [27].

Nowadays, no definitive conclusions can be drawn. Therefore, it is important to investigate cross-cultural differences in ON, OCD symptoms, core beliefs of OCD and perfectionism features, to assess the level of ON with OCD symptoms and core beliefs of OCD, and to examine the relationship between ON, OCD symptoms, core beliefs of OCD and perfectionism features.

## What your study adds?

Our study demonstrated ON’s partial independence from OCD symptoms and core beliefs of OCD in both Polish and Italian university students.
